# Association Between Newborn Metabolic Profiles and Pediatric Kidney Disease

**DOI:** 10.1016/j.ekir.2018.02.001

**Published:** 2018-02-10

**Authors:** Manish M. Sood, Malia S.Q. Murphy, Steven Hawken, Coralie A. Wong, Beth K. Potter, Kevin D. Burns, Anne Tsampalieros, Katherine M. Atkinson, Pranesh Chakraborty, Kumanan Wilson

**Affiliations:** 1 The Ottawa Hospital Research Institute, Ottawa, Ontario, Canada; 2 Institute for Clinical Evaluative Sciences, Ottawa, Ontario, Canada; 3 Clinical Epidemiology Program, University of Ottawa, Ontario, Canada; 4 Department of Medicine, University of Ottawa, Ottawa, Ontario, Canada; 5 Children’s Hospital of Eastern Ontario, Ottawa, Ontario, Canada; 6 Kidney Research Centre, University of Ottawa, Ottawa, Ontario, Canada

**Keywords:** chronic kidney disease, dialysis, end-stage kidney disease, metabolomics, newborn screening, pediatric, renal failure

## Abstract

**Introduction:**

Metabolomics offers considerable promise in early disease detection. We set out to test the hypothesis that routine newborn metabolic profiles at birth, obtained through screening for inborn errors of metabolism, would be associated with kidney disease and add incremental information to known clinical risk factors.

**Methods:**

We conducted a population-level cohort study in Ontario, Canada, using metabolic profiles from 1,288,905 newborns from 2006 to 2015. The primary outcome was chronic kidney disease (CKD) or dialysis. Individual metabolites and their ratio combinations were examined by logistic regression after adjustment for established risk factors for kidney disease and incremental risk prediction measured.

**Results:**

CKD occurred in 2086 (0.16%, median time 612 days) and dialysis in 641 (0.05%, median time 99 days) infants and children. Individual metabolites consisted of amino acids, acylcarnitines, markers of fatty acid oxidation, and others. Base models incorporating clinical risk factors only provided c-statistics of 0.61 for CKD and 0.70 for dialysis. The addition of identified metabolites to risk prediciton models resulted in significant incremental improvement in the performance of both models (CKD model: c-statistic 0.66 NRI 0.36 IDI 0.04, dialysis model: c-statistic 0.77 NRI 0.57 IDI 0.09). This was consistent after internal validation using bootstrapping and a sensitivity analysis excluding outcomes within the first 30 days.

**Conclusion:**

Routinely collected screening metabolites at birth are associated with CKD and the need for dialytic therapies in infants and children, and add incremental information to traditional clinical risk factors.

Chronic kidney disease (CKD) is a leading contributor to cardiovascular morbidity and mortality, with a global prevalence of 8% to 16% in adults. Although large population-based studies have examined the epidemiology of CKD in adult populations,^1^, ^2^, ^3^ comparable studies of CKD in children are few.^4^ The current literature suggests that 70% of children with CKD will develop end-stage kidney disease (ESKD) by age 20 years, and mortality rates for children with ESKD on dialysis therapy are 30 to 150 times higher than those in the general pediatric population.^5^, ^6^

As there are limited therapies available after kidney disease onset, early identification of individuals at risk is critical to the implementation of measures to minimize complications, to improve quality of life, and to reduce mortality. Through its role as an excretory organ the kidney plays a significant role in nutritional and metabolic regulation. Alterations in glomerular filtration, secretion, and tubular reabsorption therefore result in detectable changes in small molecule concentrations in the blood and urine. Routinely used markers of kidney function including serum creatinine and blood urea nitrogen are limited, however, by their inability to support detection of CKD in the earliest stages of the disease.

Metabolic derangements are well described in patients with CKD. Plasma and urinary amino acid profiles are demonstrably affected by acute and chronic kidney disease and by glomerulonephritis.^7^, ^8^, ^9^, ^10^, ^11^ Dysregulation of acylcarnitine excretion as a result of renal failure has also been observed in CKD and diabetic nephropathy. It is unknown whether the biological processes associated with acute illness, inflammatory processes, and kidney disease are established at the time of birth. Humans are born with a set number of functioning nephrons per kidney,^12^ and reduced nephron mass is hypothesized to underlie individual susceptibility to hypertension and CKD.^13^, ^14^, ^15^ Whereas *antemortem* measurement of nephron mass is not currently possible, metabolic profiling of circulating amino acids and acylcarnitines in the neonatal period may reveal differential renal function and susceptibility to pediatric kidney disease before clinical onset of the condition.

In this study, we set out to examine the association between routinely collected newborn metabolite profiles with development of CKD or the need for dialysis in infants and children up to 9 years of age. We hypothesized that patterns of analytes and anatlye ratios at birth would be associated with CKD or dialysis and would add incremental information to known clinical kidney disease−related risk factors.

## Methods

### Design and Setting

We conducted a population-based cohort study to determine the association between newborn metabolic profiles and the risk of CKD or dialysis. We used data collected from infants born in Ontario, Canada, through routine newborn screening and provincial outcome data from administrative databases housed at the Institute for Clinical Evaluative Sciences (ICES). The study was conducted according to a prespecified protocol with ethics approval by the Ottawa Health Science Network Research Ethics Board (20140724-01H) and the Children’s Hospital of Eastern Ontario Research Ethics Board (15/143X).

### Data Sources

Newborn metabolite data, maternal and newborn clinical data, and study outcome information were obtained by linkage between the Newborn Screening Ontario, the Better Outcomes Registry and Network, Gamma Dynacare, Canadian Organ Replacement Registry, and other ICES datasets using encrypted patient health card numbers as unique identifiers.

#### Newborn Screening Ontario

The Newborn Screening Ontario (NSO) program screens nearly all (>99%) children born in Ontario, Canada, for the presence of rare, treatable diseases using blood samples collected within the first few days of life. The newborn screening program collects data on more than 40 distinct analytes, many of which are markers of metabolism. The markers available for study from NSO are listed in Supplementary Table S1.

#### The Better Outcomes Registry and Network

The Better Outcomes Registry and Network (BORN) is a prescribed registry that includes a broad collection of prenatal and perinatal data. BORN was launched in 2012 as the integration of 5 stand-alone databases: congenital anomalies surveillance (Fetal Alert Network); pregnancy, birth, and newborn information for women in hospitals (Niday Perinatal Database); pregnancy, birth, and newborn information for women giving birth at home (Ontario Midwifery Program database); prenatal screening (Ontario Maternal Multiple Marker Serum Screening); and newborn screening (the Newborn Screening Ontario database). Data within the BORN Information System (BIS) are available to researchers for the purposes of facilitating or improving the provision of health care.

#### Institute for Clinical Evaluative Sciences

The Institute for Clinical Evaluative Sciences (ICES) houses all of Ontario’s health administrative databases. The study cohort was limited to children who were continuously registered in the Ontario Health Insurance Plan (OHIP) Claims database during the study period to ensure capture of all potential study outcomes. ICES datasets used for this study included the MOMBABY dataset, which links the admission records of delivery mothers and their newborns; the Discharge Abstract Database, which captures all administrative, clinical, and demographic information on hospital discharges; Gamma Dynacare, which captures laboratory tests; the Canadian Organ Replacement Registry, which captures all ESKD patients in Canada; and the National Ambulatory Care Registration System database, which contains data for all hospital- and community-based ambulatory care. A list of diagnostic codes used for this study is presented in Supplementary Table S2.

### Study Population

Children born between 1 April 2006 and 26 September 2015 for whom newborn screening data were available (n = 1,504,459) were included for analysis. Children for whom OHIP coverage was not continuous during the study period, cases with missing clinical data, children who died within 7 days of birth, and those who were identified as positive for one or more screened disorders in the NSO database were excluded to remove any potential outliers in the data set. Children with known or diagnosed renal dysplasia, acute kidney injury, uropathy, or urinary tract infections at birth were also excluded. In a sensitivity analysis, we further excluded all diagnoses of kidney disease listed above to 30 days after birth.

### Study Outcomes

The primary outcomes of interest were the development of CKD or the need for dialysis. CKD was defined by the use of validated International Classification of Diseases (ICD) billing codes on 2 separate days.^16^ Dialytic therapies were defined using any single validated ICD diagnostic code, an OHIP physician billing code, or a preemptive kidney transplantation.^17^, ^18^ Outcome data from Gamma Dynacare and ICES were captured up to 15 November 2016 to allow a minimum of 6 months of follow-up of the last infant included in our population subset. In this way, our analysis examined kidney outcomes 0.5 to 10 years after birth in the identified cohort.

### Statistical Analysis

Baseline characteristics of the cohort were assessed using frequency distributions and univariate descriptive statistics. Metabolite ratios were examined, as they have been previously implicated in the biological processes associated with kidney disease.^19^ A total of 46 individual metabolites and 1035 metabolite ratios were included. Metabolites and their ratios were truncated at the 0.001st percentile and the 99.999th percentile to minimize the influence of outliers, and were also standardized by study week to account for possible changes in the assays used over the study period.

To examine the association of individual metabolites with clinical outcomes we first examined crude Spearman correlations for all metabolites and their ratio combinations with each outcome of CKD or dialysis. Crude Spearman correlation magnitudes were ranked from largest to smallest to retrieve the top 100 ratios. An adjusted Spearman correlation for clinical covariates, metabolites, and the top 100 ratios were then computed, adjusting for the remaining variables. We then reduced the top-ranked metabolites or ratios to maintain 10 cases of CKD or dialysis per covariate.^20^ A mechanistic approach as opposed to an *a priori* selection of metabolites based on biochemical knowledge was used. Such an approach is advantageous because it allows for inclusion of all available data and makes no assumptions regarding underlying relationships.^7^, ^21^ We performed separate analyses for CKD and dialysis and limited the sample to 10 noncases for every case by random selection. The final model was developed using logistic regression with clinical covariates defined *a priori*. Clinical covariates included newborn sex, weight at birth, gestational age, APGAR scores, feeding status, age at sample collection, cesarean delivery, and maternal factors (smoking, diabetes, hypertension, and age at time of delivery).

Model discrimination was determined by examining the incremental improvement that the metabolite model lent to outcome prediction compared to a model consisting of perinatal and maternal covariates alone. Incremental improvement in outcome prediction was determined by examining the change in the area under the receiver operating characteristic curve (AUC), the net reclassification index (NRI), and integrated discrimination improvement (IDI).^22^ The NRI is a measure of correct reclassification of a new model compared to an old model, and IDI is a measure of the slope for model discrimination between a new and old model. Model calibration was determined by the Hosmer−Lemeshow test. The model was internally validated using bootstrapping to determine the model optimism.^23^ Internal validation was used as opposed to use of a derivation/validation study design due to the limited number of events and uniqueness of our study cohort. Model optimism was estimated as the difference between the apparent model’s performance obtained in the bootstrap sample and the actual model performance when applied to the derivation sample. The final model c-statistic was adjusted for optimism with 200 bootstrap samples performed as per simulation studies.^23^, ^24^ To avoid exclusion of subjects due to missing covariates, multiple imputation was performed prior to analysis using a Markov chain Monte Carlo algorithm (the data augmentation algorithm).^25^ Five multiple imputation datasets were generated, with all variables included in analytical models specified as predictors in the multiple imputation model. Analyses were carried out for each multiple imputation dataset and pooled across datasets using Rubin’s rules.^26^ Correlation analyses were performed using R/R Studio (RStudio Inc., Boston, MA) packages ‘rms’ and ‘Hmisc’. All remaining analyses were performed using SAS v9.4 (SAS Institute, Cary, NC).

## Results

### Cohort Characteristics

A total of 1,335,746 infants with newborn screening records were captured during the study period, of which 46,841 were excluded (11,863 screen-positive cases; 34,707 unsatisfactory samples; and 271 cases of neonatal death within 7 days of birth). The final study cohort consisted of 1,288,905 newborns, with 2086 who developed CKD and 641 who required dialysis. The median follow-up time for the total cohort was 1863 days (interquartile range [IQR], 978−2758). Median times to CKD diagnosis and dialysis were 612 days (IQR, 155−1399) and 99 days (IQR, 5−383), respectively. A summary of the cohort characteristics stratified by outcomes is presented in Table 1. Among newborns who developed CKD and required dialysis, the proportion of females was lower (CKD 43.5% vs. non-CKD 48.8%; dialysis 43.1% vs. no dialysis 48.8%), and fewer newborns were exclusively breastfed relative to the total cohort (CKD 25.3% vs. non-CKD 41.6%; dialysis 25.7% vs. no dialysis 41.6%). Kidney disease was more prevalent among infants born <37 weeks’ gestational age (CKD 18.4% vs. 7.5% non-CKD; dialysis 16.6% vs. no dialysis 7.5%), those with a lower mean APGAR score (CKD 8.01 vs. non-CKD 8.40; dialysis 7.63 vs. no dialysis 8.39), and those with a lower birthweight (CKD 3174 g vs. non-CKD 3353 g; dialysis 3132 g vs. no dialysis 3353 g). Kidney disease was more common among infants born to mothers with diabetes (CKD 17.9% vs. non-CKD 12.4%; dialysis 18.3% vs. no dialysis 12.4%) and hypertension (CKD 15.9% vs. non-CKD12.4%; dialysis 17.6% vs. no dialysis 12.4%).

**Table 1:** Baseline characteristics of the study cohort by end stage kidney disease status

Characteristic	Total	CKD	Non-CKD	*P* value	Dialysis	No dialysis	*P* value
Total, n (%)	1,288,905 (100.00)	2086 (0.16)	1,286,819 (99.84)		641 (0.05)	1,288,264 (99.95)	
Newborn							
Female, n (%)	628,881 (48.8)	907 (43.5)	627,974 (48.8)	<0.001	276 (43.1)	628,605 (48.8)	0.004
Feeding status, n (%)				<0.001			<0.001
Breast	535,612 (41.6)	527 (25.3)	535,085 (41.6)		165 (25.7)	535,447 (41.6)	
Breast/formula/TPN	112,233 (8.7)	176 (8.4)	112,057 (8.7)		75 (11.7)	112,158 (8.7)	
Formula/TPN	65,528 (5.1)	84 (4.0)	65,444 (5.1)		51 (8.0)	65,477 (5.1)	
NPO/TPN/null	78,946 (6.1)	175 (8.4)	78,771 (6.1)		98 (15.3)	78,848 (6.1)	
Gestational age, n (%)				<0.001			<0.001
≤32 wk	23,180 (1.8)	168 (8.1)	23,012 (1.8)		33 (5.1)	24,147 (1.8)	
33−36 wk	73,415 (5.7)	215 (10.3)	73,200 (5.7)		67 (10.5)	73,348 (5.7)	
≥37 wk	1,179,845 (91.5)	1687 (80.9)	1,178,158 (91.6)		534 (83.3)	1,179,311 (91.5)	
Birthweight, g, mean ± SD	3353.01 ± 568.53	3174.24 ± 795.99	3353.30 ± 568.04	<0.001	3132.66 ± 695.41	3353.12 ± 568.44	<0.001
APGAR score, mean ± SD	8.39 ± 1.32	8.01 ± 1.84	8.40 ± 1.32	<0.001	7.63± 2.08	8.39 ± 1.32	<0.001
Age at collection for newborn screening (h), median (IQR)	29.85 (24.83−44.57)	33.92 (25.40−4.43)	29.83 (24.83−44.53)	<0.001	41.77 (26.72−64.85)	29.85 (24.83−44.55)	<0.001
Maternal							
Cesarian delivery, n (%)	353,533 (27.4)	702 (33.7)	352,831 (27.4)	<0.001	198 (30.9)	353,335 (27.4)	0.136
Age at birth, yr, mean ± SD	30.17 ± 5.48	29.82 ± 5.54	30.17 ±5.48	0.004	29.98 ± 5.64	30.17 ± 5.48	0.399
Smoking, n (%)	79,918 (6.2)	162 (7.8)	79,756 (6.2)	<0.001	39 (6.1)	79,879 (6.2)	0.953
Diabetes, n (%)	160,366 (12.4)	374 (17.9)	159,992 (12.4)	<0.001	117 (18.3)	160,249 (12.4)	<0.001
Maternal hypertension, n (%)	160,209 (12.4)	332 (15.9)	159,877 (12.4)	<0.001	113 (17.6)	160,096 (12.4)	<0.001

CKD, chronic kidney disease; IQR, interquartile range; NPO, nil per os; TPN, total parenteral nutrition.

### Metabolite Models for CKD and ESKD

**Figure 1:**
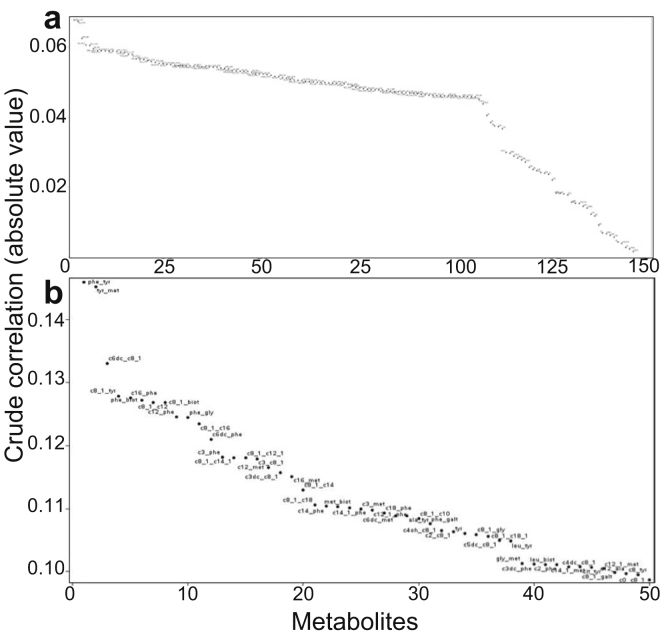
Crude Spearman correlations of metabolites in (a) chronic kidney disease and (b) dialysis.

Crude Spearman correlations for metabolites and metabolite ratios with CKD and dialysis are presented in Figure 1. For CKD, the strongest unadjusted correlations were for analyte ratios C5DC:C12, tyrosine:17-hydroxyprogesterone, phenylalanine:glycine, C5 to C14:1, and C8:1 to C12. For dialysis, the strongest correlations were for analyte ratios phenylalaine:tyrosine, tyrosine:methionine, C6DC to C8:1, C8:1 to tyrosine, and C16:phenylalanine.

The clinical variables and metabolites included in the prediction model for CKD are provided in Supplementary Table S3. Male sex (odds ratio [OR], 1.14; 95% confidence interval [CI], 1.02−1.26), feeding other than breastfeeding exclusively, APGAR score (OR, 0.96; 95% CI, 0.92−1.00), maternal diabetes (OR, 1.32; 95% CI, 1.15−1.52), and maternal age (OR, 0.98; 95% CI, 0.97−0.99) were statistically associated with CKD. CKD was also significantly associated with amino acids (citrulline), amino acid ratios (phenylalanine:glycine), acylcarnitines (C2, C4DC, C6DC, C16:1OH), acylcarnitine ratios (C4 to C12:1, C5 to C14:1, C18:1 to C18:2, C4DC to C8:1), and the ratio of amino acids and acylcarnitines to endocrine markers (alanine:17-hydroxyprogesterone, C4DC:17-hydroxyprogesterone. The strongest statistical associations were for alanine:17-hydroxyprogesterone (adjusted OR, 1.35; 95% CI, 1.07−1.70 per log unit increase), phenylalanine:glycine (adjusted OR, 1.30; 95% CI, 1.08−1.56 per log unit increase), and C4DC (adjusted OR, 0.73; 95% CI, 0.55−0.97 per log unit increase) (Table 2).

**Table 2:** Adjusted odds ratios for statistically significant newborn screening metabolites and metabolite ratios in CKD and the need for dialysis

Variable	CKD OR (95% CI)	Dialysis OR (95% CI)
Phenyalanine:glycine	1.30 (1.08–1.56)	1.43 (1.13–1.81)
Phenylalanine:tyrosine		1.51 (1.12–2.03)
Citrulline	1.11 (1.02–1.22)	
Citrullline:tyrosine		1.26 (1.11–1.43)
Alanine:17-hydroxyprogesterone	1.35 (1.07–1.70)	
C0:C8:1		0.70 (0.54–0.93)
C2	1.22 (1.02–1.45)	
C4DC:17-hydroxyprogesterone	0.79 (0.65–0.97)	
C4:C12:1	0.83 (0.70–0.97)	
C4DC:C8:1	1.14 (1.05–1.23)	
C4DC	0.73 (0.55–0.97)	
C4DC:leucine	—	
C5:C14:1	0.76 (0.64–0.92)	
C5:1:C12:1	—	
C6DC	1.14 (1.08–1.22)	
C8:tyrosine		1.14 (1.02–1.28)
C8:1:C16	—	
C8:1:C14:1		1.43 (1.14–1.79)
C8:1:C18		1.70 (1.23–2.35)
C16:phenylalanine		—
C16:1OH	1.06 (1.01–1.11)	
C18:1:C18:2	1.20 (1.02–1.40)	

CI, confidence interval; CKD, chronic kidney disease; OR, odds ratio.

**Figure 2:**
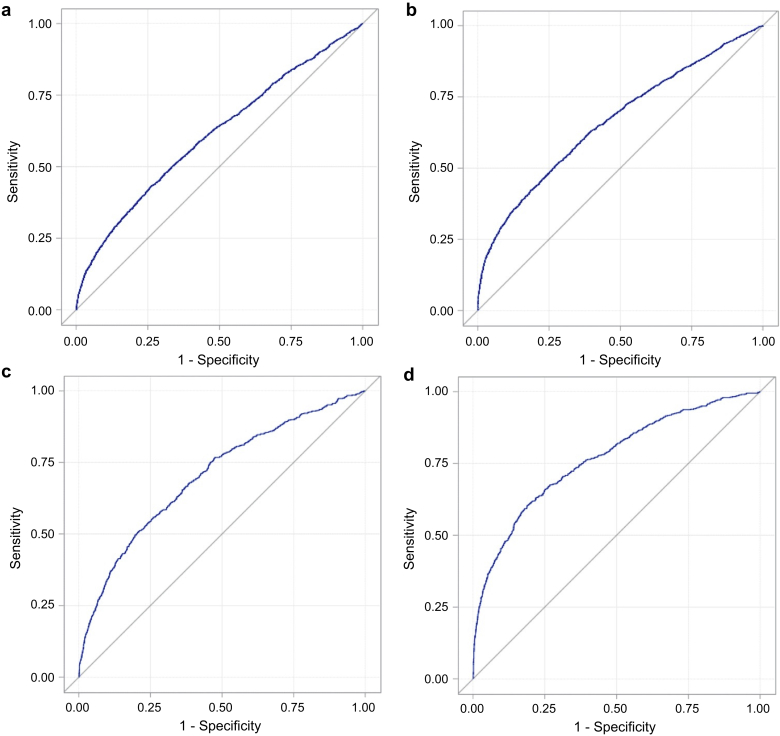
Receiver operating characteristic curves (>30 days) for the following: (a) chronic kidney disease (CKD): clinical characteristics; (b) CKD: clinical characteristics + metabolites; (c) dialysis: clinical characteristics; and (d) dialysis: clinical characteristics + metabolites.

Full models presented in Supplementary Tables S2 and S3. Models adjusted for newborn sex, birthweight, gestational age, APGAR scores, feeding status, age at sample collection, cesarean delivery, and maternal factors (smoking, diabetes, hypertension, age at time of delivery) and additional analytes identified by Spearman correlation (Figures 1 and 2).

The final model for prediction of pediatric dialysis is presented in Supplementary Table S4. Clinical covariates statistically associated with dialysis were birthweight (*P* < 0.0001), feeding other than breastfeeding exclusively, and APGAR score (OR, 0.91; 95% CI, 0.84−0.97). Need for dialysis was significantly associated with amino acid ratios (phenylalanine:glycine, phenylalanine:tyrosine, citrulline:tyrosine), ratio of amino acids to acylcarnitines (C8:tyrosine, C16:phenylalanine), and acylcarnitine ratios C0 to C8:1; C8:1 to C14:1; C8:1 to C18). The strongest metabolite associations were for C8:1 to C18:2 (adjusted OR, 1.70; 95% CI, 1.23−2.35 per log unit increase), phenylalanine:tyrosine (adjusted OR, 1.51; 95% CI, 1.12−2.03 per log unit increase), phenylalainine:glycine (adjusted OR, 1.43; 95% CI, 1.13−1.81 per log unit increase), and C8:1 to C14:1 (adjusted OR, 1.43; 95% CI, 1.14−1.79 per log unit increase) (Table 2).

### Incremental Risk Prediction of Metabolites From Traditional Clinical Risk Factors

Incremental improvements provided by newborn metabolites to CKD and dialysis risk prediction compared to known clinical risk factors are summarized in Table 3 and Figure 2a and b. For CKD, compared to a base model derived from clinical risk factors the addition of metabolites and their ratios increased the AUC from 0.61 to 0.66 (*P* < 0.001). The category-free NRI increased 0.36 (95% CI, 0.32−0.40; *P* < 0.001; 11% events correctly reclassified and 25% nonevents correctly reclassified) and the IDI increased 0.039 (95% CI, 0.034−0.044; *P* < 0.001). Model calibration was significant at *P* < 0.0001, demonstrating poor calibration. In the sensitivity analysis, AUC for the full model was 0.72 with NRI 0.49, IDI 0.024, and model calibration improved (*P* = 0.1431). After internal validation using 200 bootstrap samples the corrected AUCs were 0.64 and 0.69 for CKD and CKD 30 days after birth, respectively.

**Table 3:** Comparison of model discrimination between clinical model (base) and clinical model plus metabolites for chronic kidney disease and 30 days after birth in infants and children

Model^a^	AUC (95% CI)	*P* value for AUC difference	NRI (95% CI)	% of Events correctly reclassified	% of Nonevents correctly reclassified	IDI (95% CI)	Hosmer–Lemeshow test	AUC corrected^b^	Optimism
CKD									
Clinical risk factors Clinical risk factors + metabolites	0.61 (0.60–0.62) 0.66 (0.65–0.67)	<0.001	0.36 (0.32–0.40)	11%	25%	0.039 (0.034–0.044)	<0.001	0.64	0.018
CKD >30 days									
Clinical risk factors Clinical risk factors + metabolites	0.64 (0.61–0.67) 0.72 (0.70–0.75)	<0.001	0.49 (0.41–0.58)	23%	26%	0.024 (0.018–0.031)	0.1082	0.69	0.034

AUC, area under the receiver operating characteristic curve; CI, confidence interval; IDI, integrated discrimination improvement; NRI, net reclassification index.

a Clinical risk factors included in the model were sex, birthweight, feeding, age at sample collection, gestational age, APGAR score, and maternal cigarette smoking, hypertension, diabetes mellitus, and age at time of delivery.

b AUC corrected is based on internal validation in which optimism was calculated using 200 bootstrap samples.

Similarly, for dialysis (Table 4 and Figure 2c and d), the addition of metabolites and their ratios improved model fit (AUC changed from 0.70 to 0.77, *P* < 0.0001). The category-free NRI increased 0.57 (95% CI, 0.49−0.65; *P* < 0.001; 21% events correctly reclassified and 35% nonevents correctly reclassified) and the IDI increased 0.085 (95% CI, 0.072−0.098; *P* < 0.0001). Model calibration was acceptable (*P* = 0.1756). In the sensitivity analysis, AUC for the metabolite model was 0.72 with NRI 0.41 and IDI 0.020. After internal validation using 200 bootstrap samples, the corrected AUCs were 0.75 and 0.71 for dialysis and dialysis 30 days after birth, respectively.

**Table 4:** Comparison of model discrimination between clinical model (base) and clinical model plus metabolites for the need for dialysis and 30 days after birth in infants and children

Model^a^	AUC (95% CI)	*P* value for AUC difference	NRI (95% CI)	% of Events correctly reclassified	% of Nonevents correctly reclassified	IDI (95% CI)	Hosmer–Lemeshow test	AUC corrected^b^	Optimism
Dialysis									
Clinical risk factors Clinical risk factors + metabolites	0.70 (0.68–0.72) 0.77 (0.75–0.79)	<0.001	0.57 (0.49–0.65)	21%	35%	0.085 (0.072–0.098)	0.1756 0.1082	0.75	0.015
Dialysis >30 days									
Clinical risk factors Clinical risk factors + metabolites	0.65 (0.62–0.68) 0.72 (0.69–0.75)	<0.001	0.41 (0.31–0.51)	17%	24%	0.020 (0.015–0.026)	0.4124 0.6887	0.71	0.021

AUC, area under the receiver operating characteristic curve; CI, confidence interval; IDI, integrated discrimination improvement; NRI, net reclassification index.

a Clinical risk factors included in the model were sex, birthweight, feeding, newborn age at sample collection, gestational age, APGAR score, and maternal cigarette smoking, hypertension, diabetes mellitus, and age at time of delivery.

b AUC corrected is based on internal validation in which optimism was calculated using 200 bootstrap samples.

## Discussion

In this exploratory, population-based cohort study including data from 1,288,905 newborns, we identified an association between routinely collected newborn metabolite profiles and the development of CKD or dialysis. Models incorporating analyte and analyte ratios as covariates improved the identification of infants and children at risk for developing CKD and dialysis, beyond the use of maternal and neonatal clinical risk factors alone. Our data demonstrate that routinely collected newborn data may be used for early identification of children at risk.

Although direct cross-comparison of our findings with those of previous studies is difficult given that the majority of metabolic studies have been conducted in adult populations with established kidney disease, the metabolites used in the models described in this study have appeared previously in the literature.^27^ Among the amino acids, tyrosine and its metabolic precursor, phenylalanine, are the most consistently reported to be altered in kidney disease and were among the strongest associated metabolites in both our CKD and dialysis models. In individuals with CKD, enzyme-driven conversion of phenylalanine to tyrosine is reportedly impaired,^28^ resulting in elevated plasma phenylalanine and reduced plasma tyrosine. In our study, we detected significantly increased phenylalanine levels among newborns who later developed CKD and increases in phenylalanine/tyrosine ratios in association with the need for dialysis, consistent with previous reports. The association of CKD with changes in amino acid levels, including citrulline, glycine, and leucine, in this study also confirms the findings of others.^27^ Shah *et al.* examined metabolite profiles in nondiabetic individuals with differing CKD stages.^29^ Multiple significant metabolites that changed based on estimated glomerular filtration rate stages identified by Shah *et al.* were also identified by our approach, including ornithine, C5, and C18:2. Acylcarnitines comprised 5 of 12 and 4 of 7 of the covariates in our CKD and dialysis models, respectively, highlighting their significance in the development of kidney disease. The acylcarnitines used in our models including C0, C2, C4, C8:1, C12:1, C14:1, C16, and C16:1OH have been previously reported to be strongly associated with a decline in estimated glomerular filtration rates.^30^ Finally, production of the endocrine marker 17-hydroxyprogesterone was strongly associated with CKD and dialysis in our models. This marker has been suggested to decline in advanced CKD,^29^ although its role in the early establishment of renal disease remains unclear.

The clinical risk covariates used in our models are consistent with the known risk factors for CKD and dialysis.^14^, ^31^, ^32^ Hsu *et al.* examined maternal and prenatal risk factors for the development of CKD in 1994 children with follow-up to 20 years of age. The authors reported independent associations of low birthweight, maternal diabetes, and maternal obesity with CKD. Cataldi *et al.* reported low APGAR score and receipt of renal-toxic medications as independent risk factors for acute kidney injury in 172 preterm infants.^33^ Our study identified male sex, low birthweight, no feeding or methods aside from breastfeeding, prematurity, lower APGAR score, and maternal diabetes as independently associated with CKD or the need for dialysis. The consistency of identified risk factors between our models and previous reports strengthens our findings that the identified metabolites add significant information to clinical risk factors for kidney disease risk prediction.

Plausible mechanisms of metabolic alterations include changes in metabolites levels secondary to inflammation or oxidative stress or changes in glomerular filtration and clearance by the renal tubules.^12^, ^34^ Indeed, many characteristics identified in our models, such as low birthweight, prematurity, and low APGAR score, are associated with general illness in newborns, and, as such, many of the associated metabolites may not be kidney specific. However, it should be noted that newborns with clinically apparent kidney illness at birth were excluded, as were diagnoses within the first 30 days of life through sensitivity analyses in an attempt to isolate metabolites specifically associated with our outcomes of interest. Glomerular and tubular filtration of metabolites may be related to the nephron endowment hypothesis in which individuals born with fewer functional nephrons may be more susceptible to kidney disease with secondary insults.^35^ As there is limited glomerulogenesis beyond birth, reduced nephron mass may lead to an increase in intraglomerular pressure, glomerular hyperfiltration, and accelerated glomerulosclerosis.^12^ Indeed, a small and invasive biopsy study demonstrated roughly half the number of nephrons in men with primary hypertension.^13^ Other studies in populations at high risk for renal disease (e.g., Australian Aboriginal peoples) demonstrate an association between CKD onset with a reduction in total nephrons present at birth.^15^, ^36^ Current methods to measure nephron mass involve stereological analysis or acid maceration, and highly time-intensive and invasive procedures often performed *post mortem.*^37^ Newborn screening metabolite measurements may offer the intriguing possibility for delineation of reductions in nephron mass in the perinatal period.

Numerous novel biomarkers and metabolomics approaches are being actively sought to support the early detection of kidney disease. Metabolomics analyses can identify and quantify upwards of hundreds to thousands of small molecules in a given biological sample. Whereas the benefit of nontargeted metabolomics profiling lies in its potential for new biomarker discovery and elucidating the pathophysiological mechanisms of disease, more targeted approaches can help to distinguish CKD markers from those of similar metabolic disease states. The development, validation, and adaptation of newer methods of disease detection, although necessary, are costly and time consuming. Indeed, currently applied technologies for metabolomics analyses rely on sophisticated laboratory infrastructure, including mass spectrometry and proton nuclear magnetic resonance spectroscopy. Newborn screening is a routine public health initiative that uses internationally standardized methods for the mass spectrometric detection of inborn errors of metabolism.^38^ Here we present a novel “proof-of-concept” approach that uses existing high-quality data that are routinely captured, reliable, and widely adaptable. The early timing of measurement for newborn screening is ideal, as it facilitates disease detection in the immediate postnatal period. Leveraging newborn screening data for risk modeling approaches such as those described here could conceivably be incorporated into existing electronic medical reporting systems to complement concurrent clinical findings and be used to alert clinicians to individuals with subclinical or higher susceptibility for kidney illness.

Strengths of our study include the use of a population-level cohort with a large sample size. Current metabolomics studies are largely case-control studies in which relative measures of association may be limited by the selection of the control group. In contrast, our study was performed as a population-level analysis, thus allowing accurate relative measures of association. Here we have demonstrated that a targeted approach involving a limited number of routinely captured metabolites and their ratios provides moderate discriminative ability for identifying newborns who may develop a rare and potentially life-limiting illness. In addition, our use of a well-defined clinical outcome, dialytic therapy, with validated diagnostic and billing criteria adds to the strength of this study.^17^, ^18^, ^39^ Our approach and findings may be used to guide future work on the development of predictive models and risk scores to determine the risk of kidney disease in children. Indeed, a robust model for ESKD has recently been demonstrated to improve risk prediction, dialysis planning, and the allocation of finite resources in the adult population.^40^

Our study does have some notable limitations. Our objective was to identify the highest number of individuals with subclinical or *de novo* kidney disease, without limiting our screening to previously described biochemical pathways. By taking a mechanistic approach, as opposed to a selective approach based on biological mechanisms, our study was unable to elucidate causative mechanisms involved in the development of kidney disease in our cohort. Furthermore, despite exclusion of apparent kidney disease at birth and a sensitivity analysis excluding early diagnosis and accounting for a large number of clinical variables in our models, we lacked the serum creatinine or urinalysis data necessary to identify the presence of kidney disease. Additional limitations to this study include a limited number of maternal and newborn clinical variables available for incorporation into the base model and lack of an external validation cohort. Although validated ICD codes were used to identify CKD, the reported sensitivity is low and thus underestimates the true disease prevalence.^16^ Despite the inclusion of more than 1 million newborns, the absolute numbers of dialytic events were relatively small. We were therefore unable to distinguish the need for dialysis for acute kidney injury or ESKD.

In conclusion, our study demonstrates the association between newborn metabolite profiles and subsequent development of chronic pediatric disease. We have demonstrated the utility of routinely collected newborn metabolite profiles for the identification of infants at risk for later CKD and dialysis. Importantly, the models described in this study provide significant additional information beyond risk prediction based on traditional clinical risk factors alone. This work highlights the potential for early targeted screening, monitoring, and directing of clinical therapies toward infants at risk.

## Disclosure

All the authors declared no competing interests.

## Supplementary Materal

Table S1 Newborn screening metabolites.

Table S2 Databases and coding definitions for study outcomes and baseline characteristics.

Table S3 CKD full model including clinical characteristics and analytes identified by Spearman correlation.

Table S4 Dialysis full model including clinical characteristics and analytes identified by Spearman correlation.
