# Supplementary material for: Association Between Newborn Metabolic Profiles and Pediatric Kidney Disease
Source: Kidney Int Rep. 2018 Feb 10;3(3):691–700. doi: 10.1016/j.ekir.2018.02.001 (PMC5976820; doi:10.1016/j.ekir.2018.02.001)
Supplement: Table S1 — Newborn screening metabolites. [file mmc1.docx]

**Supplementary Table S1**: Newborn screened metabolites.

| **Marker Type** | **Marker** |
| --- | --- |
| Acyl-carnitines, Other | C0, C2, C3, C4, C5, C5:1, C6, C8, C8:1, C10, C10:1, C12, C12:1, C14, C14:1, C14:2, C16, C18, C18:1, C18:2, C3DC, C4DC, C4:OH, C5DC, C5:OH, C6DC, C14:OH, C16:OH, C16:1:OH, C18:OH, C18:1:OH |
| Amino Acids | Alanine, arginine, citrulline, glycine, leucine, methionine, ornithine, phenylalanine, tyrosine, valine |
| Enzyme/Co-Enzyme Markers | Galactose-1-Phosphate Uridyltransferase, Biotinidase, Immunotrypsinogen |
| Endocrine markers | thyroid stimulating hormone, 17-hydroxyprogesterone |
