# Supplementary material for: Association Between Newborn Metabolic Profiles and Pediatric Kidney Disease
Source: Kidney Int Rep. 2018 Feb 10;3(3):691–700. doi: 10.1016/j.ekir.2018.02.001 (PMC5976820; doi:10.1016/j.ekir.2018.02.001)
Supplement: Table S2 — Databases and coding definitions for study outcomes and baseline characteristics. [file mmc2.docx]

**Supplementary Table S2**: Databases and coding definitions for study outcomes and baseline characteristics.

| **Variable** | **Variable type** | **Database** | **Code type** | **Definition** | **Algorithm** | **Sensitivity algorithm** |
| --- | --- | --- | --- | --- | --- | --- |
| **Dialysis** | Primary definition | DAD/  NACRS | ICD10 | ‘Z992' | If present | If first occurrence ≥30 days after birth |
|  |  |  | CCI | 1PZ21HPD4' '1PZ21HQBR' '1PZ21HQBS' '1OT53DATS' '1OT53HATS' '1OT53LATS' | If present | If first occurrence ≥30 days after birth |
|  |  | OHIP | feecode | R849' 'G323' 'G325' 'G326' 'G860' 'G863' 'G866' 'G330' 'G331' 'G332' 'G861' 'G082' 'G083' 'G085' 'G090' 'G091' 'G092' 'G093' 'G094' 'G095' 'G096' 'G294' 'G295' | If present | If first occurrence ≥30 days after birth |
|  |  | CORR |  | treatment_code not in ('171' '181' '') | If present | If first occurrence ≥30 days after birth |
| **transplant** |  | DAD/NACRS | ICD10 | Z940' | If present | If first occurrence ≥30 days after birth |
| **transplant** |  | DAD/NACRS | CCI | 1PC85' | If present | If first occurrence ≥30 days after birth |
| **transplant** |  | OHIP | feecode | S435', 'S434' | If present | If first occurrence ≥30 days after birth |
| **transplant** |  | CORR |  | treatment_code in ('171') and ( transplanted_organ_type_code1 in ('10' '11' '12' '18' '19') or  transplanted_organ_type_code2 in ('10' '11' '12' '18' '19') or  transplanted_organ_type_code3 in ('10' '11' '12' '18' '19') ) | If present | If first occurrence ≥30 days after birth |
| **CKD** | Primary definition | DAD/NACRS | ICD10 | E102' 'E112' 'E132' 'E142' 'I12' 'I13' 'N08' 'N19' 'N18' | At least 2 codes on separate dates | If first occurrence ≥30 days after birth (at least 2 codes on separate dates) |
|  |  | OHIP | ICD9 | 585' '403' |  |  |
| **Kidney Disease Exclusions** | | | | | | |
| **Dysplasia** | Exclusion | DAD/  NACRS | ICD10 | Q60.0' to 'Q61.4' | If present | If first occurence <30 days after birth |
| **Acute kidney injury** | Exclusion | DAD/NACRS | ICD10 | N17' | If present | If first occurence <30 days after birth |
| **Uropathy** | Exclusion | DAD/NACRS | ICD10 | N139' | If present | If first occurence <30 days after birth |
| **Urinary tract infection** | Exclusion | DAD/NACRS | ICD10 | N390' | If present | If first occurence <30 days after birth |
| **Baseline Characteristics** | | | | | | |
| **C-section** | Mother | DAD | CCI | 5MD60 | On baby birth date admission record (admdate ≤baby bdate ≤ddate) |  |
|  | Baby | DAD | ICD10 | Z3801' 'Z3831' | On baby birth record |  |
|  | Baby | BORN |  | In BORN, if deltype = ‘2’ | On baby birth record |  |
| **Hypertension** | Mother | DAD | ICD10 | "I10" "I11" "I12" "I13" "I15" | Diagnosis before/on baby birth date |  |
|  | Mother | HYPER |  | If present in hypertension2014 (ICES extracted cohort) | Diagnosis before/on baby birth date |  |
|  | Mother | OHIP | ICD9 | "250" | Diagnosis before/on baby birth date |  |
|  | Baby | BORN |  | mathp3 | On baby birth record |  |
| **Diabetes** | Mother | DAD | ICD10 | "E10" "E11" "E12" "E13" "E14" | Diagnosis before/on baby birth date |  |
|  | Mother | DIAB |  | If present in diabetes2014 (ICES extracted cohort) | Diagnosis before/on baby birth date |  |
|  | Mother | OHIP | ICD9 | "401" "402" "403" "404" "405" | Diagnosis before/on baby birth date |  |
|  | Baby | DAD | ICD10 | P700 "P701" | On baby birth record |  |
|  | Baby | BORN |  | mathp4 mathp5 | On baby birth record |  |
| **Smoking** | Baby | BORN |  |  | On baby birth record |  |
| **Feedtype** | Baby | NSO |  |  | On baby birth record |  |
