# Supplementary material for: Association Between Newborn Metabolic Profiles and Pediatric Kidney Disease
Source: Kidney Int Rep. 2018 Feb 10;3(3):691–700. doi: 10.1016/j.ekir.2018.02.001 (PMC5976820; doi:10.1016/j.ekir.2018.02.001)
Supplement: Table S3 — CKD full model including clinical characteristics and analytes identified by Spearman correlation. [file mmc3.docx]

**Supplementary Table S3**: CKD full model including clinical characteristics and analytes identified by Spearman correlation.

| **Variable** | **Estimate** | **Standard error** | **Lower CI (mean)** | **Upper CI (mean)** | **P value** |
| --- | --- | --- | --- | --- | --- |
| Intercept | -1.751981 | 0.28494 | -2.31168 | -1.19228 | <.0001 |
| Male sex | 0.127435 | 0.053051 | 0.02345 | 0.23142 | 0.0163 |
| Gestational age |  |  |  |  |  |
| <34 weeks | reference |  |  |  |  |
| 34-36 weeks | 0.025599 | 0.170888 | -0.30935 | 0.36055 | 0.8809 |
| ≥37 weeks | -0.2414 | 0.187136 | -0.60825 | 0.12545 | 0.1971 |
| Birth weight (per gram) | 3.6222E-05 | 5.3551E-05 | -0.00007 | 0.00014 | 0.4988 |
| Feeding Status: |  |  |  |  |  |
| Breast | Referent |  |  |  |  |
| Breast/Formula/TPN | 0.346549 | 0.108709 | 0.10871 | 0.58439 | 0.0081 |
| Formula/TPN | 0.366596 | 0.120801 | 0.11038 | 0.62281 | 0.0079 |
| NPO/TPN/Null | 0.344219 | 0.106441 | 0.11816 | 0.57028 | 0.0053 |
| Age at sample collection  (per week) | 0.000128 | 0.000107 | -0.00008 | 0.00034 | 0.232 |
| APGAR Score | -0.04377 | 0.019402 | -0.08323 | -0.00431 | 0.0308 |
| C-section | 0.034984 | 0.058255 | -0.07998 | 0.14994 | 0.5489 |
| **Maternal characteristics** | | | | | |
| Cigarette smoking | -0.003384 | 0.097251 | -0.2028 | 0.19604 | 0.9725 |
| Diabetes | 0.276042 | 0.071509 | 0.13576 | 0.41633 | 0.0001 |
| Hypertension | 0.092986 | 0.073876 | -0.05188 | 0.23785 | 0.2083 |
| Maternal age | -0.016804 | 0.005079 | -0.02686 | -0.00675 | 0.0012 |
| **Newborn metabolites** | | | | | |
| c5dc_c12 | 0.041427 | 0.105219 | -0.1648 | 0.24765 | 0.6938 |
| tyr_n17p | 0.089429 | 0.077845 | -0.06361 | 0.24247 | 0.2513 |
| phe_gly | 0.261697 | 0.093127 | 0.07917 | 0.44423 | 0.005 |
| c5_c14_1 | -0.268655 | 0.0918 | -0.44858 | -0.08873 | 0.0034 |
| c8_1_c12 | -0.006423 | 0.096074 | -0.19472 | 0.18188 | 0.9467 |
| c5_c12 | 0.108216 | 0.098371 | -0.08459 | 0.30102 | 0.2713 |
| c5 | -0.034632 | 0.137393 | -0.30404 | 0.23478 | 0.801 |
| phe | 0.11343 | 0.1122 | -0.1065 | 0.33336 | 0.3121 |
| leu | -0.007663 | 0.125397 | -0.25345 | 0.23813 | 0.9513 |
| n17p | 0.134458 | 0.104989 | -0.0731 | 0.34201 | 0.2024 |
| c5dc_c14_1 | 0.01185 | 0.084253 | -0.15328 | 0.17698 | 0.8881 |
| c5dc_c12_1 | 0.132219 | 0.084812 | -0.03401 | 0.29845 | 0.119 |
| c4_c12 | 0.001863 | 0.094454 | -0.18327 | 0.18699 | 0.9843 |
| c14_1_n17p | -0.072652 | 0.106766 | -0.28286 | 0.13756 | 0.4968 |
| c10_1_c12_1 | -0.059495 | 0.077948 | -0.21227 | 0.09328 | 0.4453 |
| c12_n17p | 0.048786 | 0.10353 | -0.15458 | 0.25215 | 0.6377 |
| c10_1_c12 | 0.033575 | 0.091897 | -0.14654 | 0.21369 | 0.7148 |
| n17p_biot | 0.046965 | 0.097483 | -0.14449 | 0.23842 | 0.6301 |
| c5_c12_1 | 0.101712 | 0.087302 | -0.0694 | 0.27282 | 0.244 |
| c8_1_c12_1 | -0.09994 | 0.081984 | -0.26063 | 0.06075 | 0.2228 |
| c8_1_c10 | 0.073478 | 0.069257 | -0.06227 | 0.20922 | 0.2887 |
| gly_n17p | 0.081503 | 0.132203 | -0.1779 | 0.3409 | 0.5377 |
| n17p_galt | 0.002974 | 0.070452 | -0.13694 | 0.14289 | 0.9664 |
| c16_n17p | -0.010068 | 0.144794 | -0.29405 | 0.27392 | 0.9446 |
| c8_c12 | -0.037508 | 0.08806 | -0.2101 | 0.13509 | 0.6702 |
| c8_1_c14_1 | 0.152864 | 0.093053 | -0.02952 | 0.33524 | 0.1004 |
| c4dc_n17p | -0.234825 | 0.101649 | -0.43527 | -0.03438 | 0.0219 |
| c5_gly | -0.064013 | 0.118032 | -0.29537 | 0.16734 | 0.5876 |
| c5_c16 | -0.168327 | 0.106885 | -0.37782 | 0.04116 | 0.1153 |
| c5_galt | -0.157729 | 0.086248 | -0.32877 | 0.01331 | 0.0703 |
| c4_c4oh | 0.084003 | 0.047552 | -0.0092 | 0.17721 | 0.0773 |
| c12_1_n17p | -0.053185 | 0.092259 | -0.23634 | 0.12997 | 0.5657 |
| c5dc_c16 | 0.038439 | 0.090845 | -0.13961 | 0.21649 | 0.6722 |
| c4_c14_1 | 0.082476 | 0.089381 | -0.09271 | 0.25766 | 0.3561 |
| c5dc_c10 | -0.032844 | 0.068809 | -0.16771 | 0.10202 | 0.6331 |
| c5_biot | 0.077676 | 0.105935 | -0.13011 | 0.28546 | 0.4635 |
| c12_phe | 0.21484 | 0.174326 | -0.12684 | 0.55652 | 0.2178 |
| c4_c12_1 | -0.190471 | 0.082273 | -0.35172 | -0.02922 | 0.0206 |
| c10_n17p | -0.00432 | 0.085333 | -0.17252 | 0.16388 | 0.9597 |
| c18_2_galt | 0.012292 | 0.068119 | -0.12122 | 0.14581 | 0.8568 |
| c4dc_phe | 0.187772 | 0.13264 | -0.0722 | 0.44775 | 0.1569 |
| c4dc_c5 | 0.03321 | 0.065482 | -0.09514 | 0.16156 | 0.6121 |
| c18_2_biot | -0.010432 | 0.101074 | -0.20879 | 0.18792 | 0.9178 |
| c4_c4dc | 0.025504 | 0.078325 | -0.12801 | 0.17902 | 0.7447 |
| c8_c12_1 | 0.00238 | 0.076714 | -0.14798 | 0.15274 | 0.9752 |
| c4_c16 | 0.121558 | 0.098608 | -0.07171 | 0.31483 | 0.2177 |
| c5_c10 | 0.033276 | 0.07455 | -0.11284 | 0.17939 | 0.6553 |
| c8_c14_1 | -0.025688 | 0.083617 | -0.18957 | 0.1382 | 0.7587 |
| c8_1_c16 | -0.110929 | 0.104027 | -0.31482 | 0.09296 | 0.2863 |
| c10_1_c14_1 | -0.016179 | 0.086645 | -0.186 | 0.15364 | 0.8519 |
| c14oh_n17p | -0.015885 | 0.073289 | -0.15999 | 0.12822 | 0.8285 |
| c5_c18 | 0.091865 | 0.085626 | -0.07596 | 0.25969 | 0.2833 |
| c18oh_n17p | -0.035216 | 0.065746 | -0.16423 | 0.09379 | 0.5923 |
| c4_c10 | 0.019901 | 0.075137 | -0.12737 | 0.16717 | 0.7911 |
| c4oh_c5 | 0.061495 | 0.068274 | -0.07232 | 0.19531 | 0.3678 |
| c4oh_n17p | 0.089656 | 0.088693 | -0.08441 | 0.26372 | 0.3124 |
| c0_c12 | 0.106147 | 0.111591 | -0.11257 | 0.32486 | 0.3415 |
| leu_gly | -0.057707 | 0.104673 | -0.26287 | 0.14745 | 0.5814 |
| c4dc_c8_1 | 0.127268 | 0.040795 | 0.04731 | 0.20722 | 0.0018 |
| c14_n17p | 0.02497 | 0.125084 | -0.22462 | 0.27456 | 0.8424 |
| c4dc_leu | -0.250375 | 0.141661 | -0.52803 | 0.02728 | 0.0772 |
| c18_n17p | -0.163853 | 0.147406 | -0.4536 | 0.1259 | 0.267 |
| c4dc_c18_2 | 0.013193 | 0.074489 | -0.1328 | 0.15919 | 0.8594 |
| c8_1 | -0.082242 | 0.128614 | -0.33456 | 0.17008 | 0.5226 |
| c10_1 | -0.038046 | 0.10835 | -0.25045 | 0.17435 | 0.7255 |
| c18_2 | 0.019777 | 0.112639 | -0.20105 | 0.2406 | 0.8606 |
| val | 0.114073 | 0.070295 | -0.0237 | 0.25185 | 0.1046 |
| c8_1_gly | 0.126598 | 0.103146 | -0.07557 | 0.32876 | 0.2197 |
| c5_tyr | 0.118336 | 0.063945 | -0.00699 | 0.24367 | 0.0642 |
| c12_leu | 0.126013 | 0.165797 | -0.19895 | 0.45097 | 0.4472 |
| c5oh_c12 | -0.037156 | 0.07372 | -0.18164 | 0.10733 | 0.6142 |
| c14_1_phe | 0.073825 | 0.17877 | -0.27656 | 0.42421 | 0.6796 |
| c16_phe | 0.023346 | 0.134624 | -0.24052 | 0.28721 | 0.8623 |
| c5_c14 | 0.09439 | 0.100107 | -0.10182 | 0.2906 | 0.3457 |
| c4dc_c5dc | 0.168718 | 0.082255 | 0.00749 | 0.32994 | 0.0403 |
| c8_1_biot | 0.0515 | 0.107407 | -0.16235 | 0.26535 | 0.6329 |
| phe_galt | 0.06177 | 0.104762 | -0.14667 | 0.27021 | 0.5571 |
| c3dc_c12 | -0.014834 | 0.09827 | -0.20744 | 0.17777 | 0.88 |
| leu_biot | -0.056995 | 0.113683 | -0.28028 | 0.16629 | 0.6163 |
| c3dc_c5dc | -0.036995 | 0.073288 | -0.18064 | 0.10665 | 0.6137 |
| phe_biot | -0.076663 | 0.116921 | -0.30641 | 0.15308 | 0.5123 |
| c4dc_val | 0.249512 | 0.132428 | -0.01004 | 0.50907 | 0.0595 |
| orn_n17p | -0.077372 | 0.098396 | -0.27024 | 0.1155 | 0.4317 |
| c8_c16 | -0.070999 | 0.092258 | -0.25182 | 0.10982 | 0.4416 |
| c8_1_galt | -0.00491 | 0.084981 | -0.17439 | 0.16457 | 0.9541 |
| c5_c18_1 | 0.128562 | 0.089365 | -0.04659 | 0.30372 | 0.1503 |
| c5_ala | 0.053697 | 0.083287 | -0.10955 | 0.21694 | 0.5191 |
| c10_1_biot | 0.021543 | 0.10563 | -0.1873 | 0.23038 | 0.8387 |
| c2_c12 | -0.064859 | 0.11054 | -0.28151 | 0.15179 | 0.5574 |
| c10_1_galt | -0.023086 | 0.082962 | -0.18807 | 0.14189 | 0.7815 |
| c14_1_leu | -0.014362 | 0.144277 | -0.29714 | 0.26842 | 0.9207 |
| c10_1_c16 | 0.150832 | 0.095573 | -0.03649 | 0.33815 | 0.1145 |
| c18_1oh_n17p | 0.015377 | 0.068831 | -0.11999 | 0.15075 | 0.8234 |
| c14_1_c18_2 | -0.20886 | 0.083881 | -0.37327 | -0.04445 | 0.0128 |
| leu_galt | 0.097065 | 0.101534 | -0.10317 | 0.2973 | 0.3403 |
| c0_c4dc | 0.015591 | 0.071068 | -0.1237 | 0.15488 | 0.8264 |
| ala_n17p | 0.29821 | 0.11849 | 0.06597 | 0.53045 | 0.0118 |
| c18_1_n17p | 0.116925 | 0.143182 | -0.16372 | 0.39757 | 0.4142 |
| c8_1_c14 | 0.063591 | 0.094977 | -0.12256 | 0.24974 | 0.5032 |
| c12_cit | 0.00996 | 0.077465 | -0.14187 | 0.16179 | 0.8977 |
| c5_1_c12_1 | 0.063927 | 0.053609 | -0.04115 | 0.169 | 0.2331 |
| c18_2_tyr | 0.034287 | 0.067739 | -0.09848 | 0.16705 | 0.6127 |
| c4_c14 | -0.075233 | 0.100327 | -0.27187 | 0.12141 | 0.4533 |
| c6_n17p | -0.059423 | 0.072084 | -0.20079 | 0.08194 | 0.4098 |
| c12_1_phe | -0.298611 | 0.164273 | -0.62058 | 0.02336 | 0.0691 |
| c18_1_c18_2 | 0.180772 | 0.080143 | 0.02369 | 0.33785 | 0.0241 |
| c10_c10_1 | -0.013638 | 0.044392 | -0.10065 | 0.07337 | 0.7587 |
| c12_val | -0.13815 | 0.136842 | -0.40636 | 0.13006 | 0.3127 |
| c4 | -0.059693 | 0.08917 | -0.23446 | 0.11508 | 0.5032 |
| c4dc | -0.311231 | 0.143998 | -0.59348 | -0.02898 | 0.0307 |
| c5dc | 0.135709 | 0.107959 | -0.07589 | 0.34731 | 0.2087 |
| c5oh | 0.058779 | 0.054933 | -0.04889 | 0.16645 | 0.2846 |
| c8 | 0.087224 | 0.07391 | -0.05764 | 0.23209 | 0.2379 |
| c12 | -0.233456 | 0.173976 | -0.57446 | 0.10755 | 0.1796 |
| c12_1 | 0.322052 | 0.165268 | -0.00191 | 0.64601 | 0.0514 |
| c14_1 | -0.057807 | 0.176286 | -0.40333 | 0.28772 | 0.743 |
| c14_2 | -0.02852 | 0.026742 | -0.08093 | 0.02389 | 0.2862 |
| c16 | 0.059195 | 0.147447 | -0.2298 | 0.34819 | 0.6881 |
| arg | 0.001433 | 0.029623 | -0.05663 | 0.0595 | 0.9614 |
| cit | 0.107921 | 0.046873 | 0.01605 | 0.19979 | 0.0213 |
| tyr | -0.032323 | 0.054645 | -0.13955 | 0.0749 | 0.5543 |
| asa | 0.043407 | 0.024938 | -0.00547 | 0.09229 | 0.0818 |
| met | -0.061248 | 0.032077 | -0.12414 | 0.00164 | 0.0563 |
| tsh | -0.002269 | 0.027402 | -0.05598 | 0.05144 | 0.934 |
| c0 | -0.162092 | 0.096105 | -0.35046 | 0.02627 | 0.0917 |
| c2 | 0.196565 | 0.090772 | 0.01865 | 0.37448 | 0.0304 |
| c3 | -0.070431 | 0.033603 | -0.13629 | -0.00457 | 0.0361 |
| c3dc | 0.121136 | 0.098283 | -0.0715 | 0.31377 | 0.2178 |
| c4oh | -0.14447 | 0.078545 | -0.29842 | 0.00948 | 0.0659 |
| c5_1 | -0.010987 | 0.040655 | -0.09067 | 0.0687 | 0.787 |
| c6 | -0.004899 | 0.050177 | -0.10326 | 0.09346 | 0.9222 |
| c6dc | 0.135186 | 0.031543 | 0.07336 | 0.19701 | <.0001 |
| c10 | -0.020117 | 0.073117 | -0.16349 | 0.12326 | 0.7832 |
| c14 | 0.077419 | 0.084759 | -0.08924 | 0.24408 | 0.3616 |
| c14oh | -0.027923 | 0.053564 | -0.13312 | 0.07727 | 0.6024 |
| c16oh | 0.033438 | 0.027159 | -0.01979 | 0.08667 | 0.2183 |
| c16_1oh | 0.057331 | 0.026001 | 0.00637 | 0.10829 | 0.0275 |
| c18 | 0.06163 | 0.09371 | -0.12211 | 0.24537 | 0.5108 |
| c18_1 | -0.079708 | 0.098501 | -0.27278 | 0.11336 | 0.4184 |
| c18oh | -0.006357 | 0.048827 | -0.10213 | 0.08942 | 0.8964 |
| c18_1oh | -0.050411 | 0.049937 | -0.14851 | 0.04769 | 0.3132 |
| ala | -0.107898 | 0.075646 | -0.25616 | 0.04037 | 0.1538 |
| orn | -0.02569 | 0.054118 | -0.13177 | 0.08039 | 0.635 |
| gly | 0.124393 | 0.091189 | -0.05434 | 0.30313 | 0.1725 |
| biot | -0.048862 | 0.065218 | -0.17717 | 0.07944 | 0.4543 |
| galt | -0.005119 | 0.063906 | -0.13527 | 0.12504 | 0.9367 |
