# Supplementary material for: Association Between Newborn Metabolic Profiles and Pediatric Kidney Disease
Source: Kidney Int Rep. 2018 Feb 10;3(3):691–700. doi: 10.1016/j.ekir.2018.02.001 (PMC5976820; doi:10.1016/j.ekir.2018.02.001)
Supplement: Table S4 — Dialysis full model including clinical characteristics and analytes identified by Spearman correlation. [file mmc4.docx]

**Supplementary Table S4**: Dialysis full model including clinical characteristics and analytes identified by Spearman correlation.

| **Variable** | **Estimate** | **Standard error** | **Lower CI(mean)** | **Upper CI (mean)** | **P value** |
| --- | --- | --- | --- | --- | --- |
| Intercept | -0.89507 | 0.47239 | -1.82109 | 0.03094 | <.0001 |
| Sex (male) | 0.099171 | 0.095641 | -0.08831 | 0.28665 | 0.8707 |
| Gestational age: |  |  |  |  |  |
| <34 weeks | Referent |  |  |  |  |
| 34-36 weeks | 1.077514 | 0.331538 | 0.42669 | 1.72834 | 0.0638 |
| ≥37 weeks | 1.106349 | 0.338105 | 0.44306 | 1.76963 | 0.0448 |
| Birth weight (per gram) | -0.00035 | 9.62E-05 | -0.00054 | -0.00016 | 0.0005 |
| Feeding Status |  |  |  |  |  |
| Breast | Reference |  |  |  |  |
| Breast/Formula/TPN | 0.581384 | 0.155434 | 0.26667 | 0.8961 | 0.0136 |
| Formula/TPN | 0.90133 | 0.202717 | 0.4735 | 1.32916 | 0.0049 |
| NPO/TPN/Null | 0.927479 | 0.202057 | 0.48709 | 1.36787 | 0.0384 |
| Age at sample collection (per week) | 0.000176 | 0.000172 | -0.00016 | 0.00051 | 0.787 |
| APGAR | -0.20734 | 0.028128 | -0.26267 | -0.15201 | 0.0077 |
| C-section | -0.12902 | 0.109596 | -0.34507 | 0.08703 | 0.95 |
| **Maternal characteristics** | | | | | |
| Cigarette smoking | -0.16122 | 0.231499 | -0.6652 | 0.34275 | 0.5629 |
| Diabetes | 0.332594 | 0.138571 | 0.06022 | 0.60497 | 0.0732 |
| Hypertension | 0.119193 | 0.136889 | -0.14963 | 0.38801 | 0.2067 |
| Maternal age | -0.01085 | 0.009341 | -0.02929 | 0.00759 | 0.1132 |
| **Newborn Metabolites** | | | | | |
| phe_tyr | 0.40987 | 0.152983 | 0.11002 | 0.70972 | 0.4945 |
| tyr_met | 0.164377 | 0.137781 | -0.10575 | 0.43451 | 0.5966 |
| c6dc_c8_1 | -0.12317 | 0.157597 | -0.4321 | 0.18576 | 0.3666 |
| c8_1_tyr | 0.0997 | 0.144421 | -0.1835 | 0.3829 | 0.5686 |
| c16_phe | -0.344 | 0.219243 | -0.77385 | 0.08585 | 0.0377 |
| phe_biot | 0.06866 | 0.163542 | -0.25219 | 0.38951 | 0.2563 |
| c8_1_c12 | -0.22251 | 0.127508 | -0.47242 | 0.0274 | 0.0962 |
| c8_1_biot | -0.2176 | 0.159038 | -0.53543 | 0.10023 | 0.2527 |
| c12_phe | -0.3523 | 0.237986 | -0.81878 | 0.11419 | 0.3567 |
| phe_gly | 0.355648 | 0.1206 | 0.11927 | 0.59202 | 0.8603 |
| c8_1_c16 | -0.2946 | 0.171833 | -0.63139 | 0.0422 | 0.2053 |
| c6dc_phe | 0.118662 | 0.192692 | -0.25904 | 0.49636 | 0.6007 |
| c3_phe | 0.109439 | 0.196623 | -0.27599 | 0.49487 | 0.1916 |
| c8_1_c14_1 | 0.357438 | 0.115058 | 0.13191 | 0.58296 | 0.0018 |
| c8_1_c12_1 | -0.00302 | 0.090611 | -0.18063 | 0.17458 | 0.9128 |
| c3_c8_1 | 0.105384 | 0.151645 | -0.19184 | 0.40261 | 0.2942 |
| c12_met | 0.22697 | 0.246474 | -0.25614 | 0.71008 | 0.2492 |
| c3dc_c8_1 | -0.10794 | 0.191053 | -0.48239 | 0.26652 | 0.3096 |
| c16_met | 0.303816 | 0.213228 | -0.11415 | 0.72179 | 0.1058 |
| c8_1_c14 | -0.12589 | 0.14868 | -0.4173 | 0.16552 | 0.2041 |
| c8_1_c18 | 0.531773 | 0.165128 | 0.20812 | 0.85542 | 0.11 |
| c14_phe | -0.02597 | 0.114753 | -0.25088 | 0.19895 | 0.8511 |
| met_biot | 0.275278 | 0.147108 | -0.01335 | 0.56391 | 0.9737 |
| c14_1_phe | 0.435019 | 0.2266 | -0.00914 | 0.87918 | 0.4459 |
| c3_met | -0.35115 | 0.198343 | -0.73993 | 0.03763 | 0.0582 |
| c12_1_phe | 0.050009 | 0.246664 | -0.43355 | 0.53357 | 0.4394 |
| c18_phe | 0.169847 | 0.119847 | -0.06512 | 0.40481 | 0.2847 |
| c6dc_met | -0.21622 | 0.201058 | -0.61029 | 0.17786 | 0.7902 |
| ala_tyr | -0.07411 | 0.120272 | -0.30984 | 0.16163 | 0.2197 |
| c8_1_c10 | -0.00357 | 0.064362 | -0.12972 | 0.12258 | 0.6502 |
| phe_galt | 0.083907 | 0.092444 | -0.09785 | 0.26567 | 0.9484 |
| tyr | 0.170854 | 0.106561 | -0.03803 | 0.37974 | 0.5256 |
| c4oh_c8_1 | 0.175023 | 0.094223 | -0.00986 | 0.35991 | 0.1875 |
| c2_c8_1 | 0.216454 | 0.207334 | -0.18997 | 0.62288 | 0.0554 |
| c8_1_gly | 0.191544 | 0.162341 | -0.12687 | 0.50995 | 0.0862 |
| c8_1_c18_1 | -0.05201 | 0.117509 | -0.28233 | 0.17832 | 0.9359 |
| c5dc_c8_1 | 0.168306 | 0.132549 | -0.09149 | 0.4281 | 0.2426 |
| leu_tyr | -0.02885 | 0.13427 | -0.29235 | 0.23465 | 0.4252 |
| gly_met | 0.239306 | 0.141154 | -0.03736 | 0.51597 | 0.771 |
| c3dc_phe | 0.003128 | 0.108928 | -0.21037 | 0.21663 | 0.7569 |
| c2_phe | 0.030068 | 0.115394 | -0.19627 | 0.2564 | 0.4332 |
| leu_biot | 0.015416 | 0.12272 | -0.22553 | 0.25636 | 0.4828 |
| c14_1_met | -0.16317 | 0.241011 | -0.63555 | 0.30921 | 0.8983 |
| c4dc_c8_1 | 0.021449 | 0.086683 | -0.14845 | 0.19135 | 0.6299 |
| cit_tyr | 0.232485 | 0.065149 | 0.10479 | 0.36018 | 0.0143 |
| c12_1_met | -0.10422 | 0.257931 | -0.60988 | 0.40143 | 0.277 |
| c8_1_galt | 0.058718 | 0.124162 | -0.18537 | 0.30281 | 0.8022 |
| c12_ala | -0.14668 | 0.147382 | -0.43557 | 0.14221 | 0.1442 |
| c8_tyr | 0.131023 | 0.058661 | 0.01603 | 0.24602 | 0.4062 |
| c0_c8_1 | -0.35109 | 0.139737 | -0.62497 | -0.07721 | <.0001 |
